# The Acidogenicity of Various Chocolates Available in Indian Market: A Comparative Study

**DOI:** 10.5005/jp-journals-10005-1025

**Published:** 2009-08-26

**Authors:** Amitha M Hegde, Rajmohan Shetty, Aletta Reema Sequeira

**Affiliations:** 1 Professor and Head, Department of Pedodontics and Preventive Children Dentistry, AB Shetty Memorial Institute of Dental Sciences, Mangalore, Karnataka, India; 2 Professor, Department of Pedodontics and Preventive Children Dentistry, AB Shetty Memorial Institute of Dental Sciences Mangalore, Karnataka, India; 3 Postgraduate Student, Department of Pedodontics and Preventive Children Dentistry, AB Shetty Memorial Institute of Dental Sciences, Mangalore, Karnataka, India

**Keywords:** Unfilled chocolates, filled chocolates, pH, plaque.

## Abstract

It is widely accepted that all foods containing "fermentable carbohydrates" have the potential to contribute to caries formation. Fermentable carbohydrates are present in most starches and all sugars, including those that occur naturally in foods and those added in processed foods. The relative cariogenicity of chocolates is dependent on their composition, texture, solubility, retentiveness and ability to stimulate salivary flow. The composition of the chocolates has profound impact on its cariogenic potential. There are a wide range of chocolates available in the market and very few studies have compared the chocolates available in the Indian market.

This study was an *in vivo* study done on 30 dental volunteers where the cariogenicity between filled and unfilled chocolates were compared by evaluating the pH of plaque at different time intervals taken at baseline and at 5, 10, 15, 20 and 30 minutes using a pH meter. In unfilled group, milk chocolate had maximum pH drop at 20 minutes (5.895) and diet chocolate had minimum pH drop at 10 minutes (6.143). In filled group, fruit and nut had maximum pH drop at 20 minutes (5.713) and caramel had minimum pH drop at 15 minutes (5.817). The results between unfilled and filled chocolate were found to be statistically significant between 15-30 minutes (p < 0.0005) and suggestive that filled chocolates were more cariogenic than unfilled chocolates.

## INTRODUCTION

The science of dentistry has existed for long, ever since there has been theorizing about the cause of dental caries. Today all experts generally agree that dental caries is an infectious and communicable disease and that multiple factors influence the initiation and progression of the disease.^1^

The interaction between chocolate and dental caries has been evaluated by using several methodologies available to assess the relationship between diet and dental caries.^2^ It is widely accepted that all foods containing "fermentable carbohydrates" have the potential to contribute to caries formation.^3^

The majority of plaque pH studies have compared one type of chocolate with the other commonly consumed products and assumptions were made about their relative cariogenicity.^2^ Accordingly, it was revealed that although chocolate is among the foods that are considered as cariogenic, chocolate’s cariogenicity might be low to moderate.^2^

Also, there are only few studies where the different types of chocolates have been studied whereby, mainly milk and plain (dark) chocolates have been compared with a number of other foods, mainly snacks, or were used as products with known cariogenic potential in order to compare other foods.^2^

There are some other factors that should be considered regarding the cariogenic potential of chocolates. Different regulations regarding chocolate manufacturing, constituents, and definitions exist between various countries.^4^,^5^ Cocoa and its extracts, which have been reported to exhibit an anticariogenic action is one such constituent used in different proportions and only in some cases have these proportions been studied for any relationship to plaque acidogenicity.^6^,^7^ Additionally, cocoa levels in chocolate seem to be related to the percentages of the other constituents, such as carbohydrates that might also influence the cariogenic potential. It was revealed that different cocoa proportions in chocolate confectionery might be a factor influencing the cariogenic response of different types and their acidogenic potential was considered worthy for study.^2^ The purpose of this study was to assess the acidogenic response of plaque to chocolates with varying cocoa contents, those containing hazelnuts and diet chocolate.

## MATERIAL AND METHODS

30 volunteers who reported to the Department of Pedodontics and Preventive Children Dentistry, AB Shetty Memorial Institute of Dental Sciences, Mangalore were included in the study.

Six commercially available chocolates in the Indian market were divided into two subgroups, unfilled and filled. Plain milk, dark and diet chocolates came under the unfilled chocolates.

Chocolates with fruits and nuts, caramel and coconut were used under the group of filled chocolates. The quantity of each chocolate tested was 15 gm.

## PLAQUE SAMPLING (HARVESTING) TECHNIQUE AND pH MEASUREMENTS

Plaque pH was measured using the technique of Fosdick et al,^8^ later modified by Frostell J^9^ and Rugg-Gunn et al.^10^ Subjects participating in the study were asked to refrain from toothbrushing at least for 48 hours and from eating or drinking (apart from water) at least 2.5 hours prior to each visit.^2^ On each of the test days, pooled plaque samples of approximately 1mg were removed from six buccal surfaces, of posterior teeth representing all the quadrants of the mouth, using a sterile blunt explorer.^2^ Each plaque sample was thoroughly mixed with 20 ml of distilled water, measured by a pipette into a disposable tray and carried with another pipette into the pH system for recording. The reading was recorded and thereafter, the electrode was cleaned with a stream of distilled water and dried. The electrode was calibrated before starting the tests and in between measurements by using two buffering solutions of pH 4.0 and 7.0.^2^

A plaque sample taken before the test products were consumed and a baseline plaque pH was recorded portable standard digital pH meter with glass microelectrode, model {Eq- 612 with stand for the pH electrode (Elicoelectronics, Mumbai)}. The subjects were then instructed to eat the chocolates. Plaque samples were taken at baseline and at 5, 10, 15, 20, and 30 minutes thereafter for the measurement of the plaque pH.

All the data obtained were statistically evaluated using Anova.

## RESULTS

**Graph 1:**
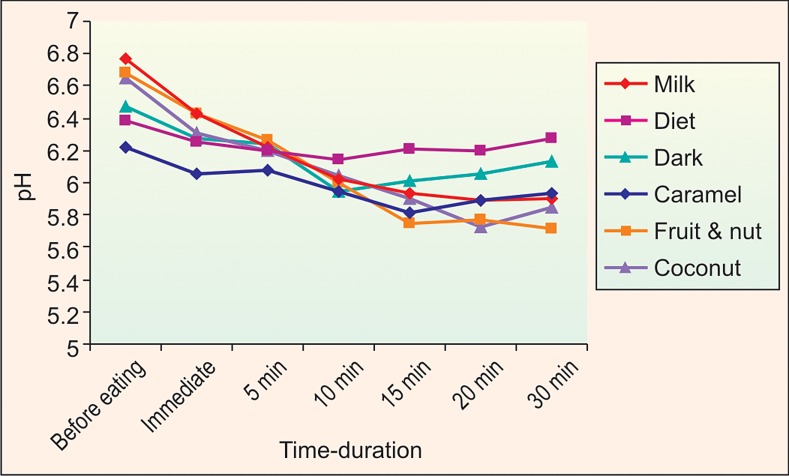
pH changes of saliva with unfilled and filled at different time intervals

The plaque pH for all the unfilled chocolates was determined. It is seen that the pH with respect to all the three chocolates dip immediately after the consumption of the chocolates and continue to do so until 10-15 minutes after which the pH is seen to raise to reach almost its initial levels (Graph 1). The drop in pH between 5-10 is not statistically significant, but the pH changes seen from 15- 30 minutes after consumption was statistically significant (Table 1).

It was found that the maximum drop of pH was seen with respect to the milk chocolate at 20 minutes (5.895) and dark chocolate at 10 minutes (5.947) while the least was with the diet chocolate at 10 minutes (6.143), suggesting diet chocolate was less cariogenic in the unfilled group (Graph 1).

Similarly, the plaque pH for filled chocolates was evaluated, it showed that all the chocolates in the group showed a fall in the levels of pH and it was more significant with chocolates with fruits and nuts than chocolates with coconut and caramel.

It was found that the maximum drop of pH was seen with the fruit and nut chocolate at 20 minutes (5.713), with coconut chocolate at 20 minutes (5.720) and the least was with the caramel at 15 minutes (5.817), suggesting caramel chocolate to be less cariogenic in the filled group (Graph 1).

When the chocolates of both the groups were compared, it was seen that the plaque pH between 5-10 minutes, there was no significant difference, but between 15-30 minutes it is statistically significant (p < 0.0005). Fruit and nut chocolate had the maximum fall in plaque pH making it most cariogenic followed closely by the coconut chocolate and the least fall in plaque pH was recorded with diet chocolate making it least cariogenic (Table 1 and Graph 1).

## DISCUSSION

Chocolate has always been associated with dental caries in various literatures both positively and negatively, although yet nothing of great significance has been proved for or against chocolates.^2^ In this study, adults are used as test subjects, while the results are reported for their applicability to children. Previously, authors have compared plaque pH findings made on both adults and children when using the same test materials and it was found that the response in children was less than in adults. Therefore, any results of plaque pH response will be greater than in children but suitable adult subjects are easier to find than children who at times can be uncooperative. In this study, the findings on adults are therefore directly applicable on children.^8^

Results from various studies have been presented in different forms, such as using minimum pH, time and areas under given pH values. Clearly, a substrate that does not cause any drop in plaque pH will probably have no detrimental effects on teeth substance. The volume of time that pH remains depressed is important and the time spent under different pH values is probably indicative of any foods retentiveness and may definitely have an effect on its cariogenicity.^9^ The ‘critical pH’ or the pH at which enamel begins to dissolve is not known though widely assumed. The critical pH varies among individuals and among oral sites within an individual and it has also been noted that the dissolution of enamel is a result of what happens in the plaque, pellicle and enamel.^9^,^10^

**Table 1:** Table 1: Comparing the mean values of pH changes of saliva with unfilled and filled chocolates

*Mean value*		*Before eating*		*Immediate*		*5 minutes*		*10 minutes*		*15 minutes*		*20 minutes*		*30 minutes*
Milk		6.772		6.425		6.217		6.027		5.930		5.895		5.899
Diet		6.386		6.249		6.307		6.143		6.207		6.203		6.280
Dark		6.473		6.275		6.243		5.947		6.011		6.050		6.129
Caramel		6.215		6.053		6.433		5.947		5.817		5.893		5.937
Fruit and nut		6.677		6.076		6.263		6.003		5.750		5.713		5.767
Coconut		6.650		6.197		6.180		6.042		5.903		5.720		5.843
Total		6.529		6.290		6.199		6.018		5.936		5.921		5.967
*Standard Deviation*														
Milk		0.3448		0.3506		0.3668		0.3258		0.2087		0.2002		0.2979
Diet		0.4482		0.3498		0.3275		0.3115		0.2864		0.2871		0.2941
Dark		0.2516		0.2153		0.2515		0.1943		0.3125		0.2840		0.2443
Caramel		0.2502		0.2006		0.3324		0.1943		0.1724		0.2132		0.2205
Fruit and nut		0.4352		0.4037		0.3499		0.2539		0.3432		0.1807		0.1807
Coconut		0.2428		0.2318		0.2748		0.2464		0.3285		0.1789		0.2712
Total		0.3859		0.3243		0.3209		0.2644		0.3149		0.2791		0.3119
P-value		0.0005		0.0005		0.279		0.040		0.0005		0.0005		0.0005

Different evaluations of ‘critical pH’ have varied from 5.7-5.5 or even lower. Prolonged drop of pH is considered to be much more harmful than that of short duration. Additionally the depth of the pH drop within a certain period of time is proportional to the potential dissolution of enamel.^2^

In this study, when unfilled chocolate was compared milk chocolate had maximum pH drop. It has been suggested that when milk is added to the chocolate to make milk chocolates, the milk may cancel out the beneficial properties of the cocoa mass which is seen in all the chocolates and can make it more cariogenic than the rest.^11^

The dark chocolates also showed a steady fall in the levels of pH but comparatively less cariogenic when compared to the milk chocolates. The lesser cariogenicity of dark chocolates may be because of the fact that dark chocolate boost the antioxidant levels and also have higher concentrations of unsaturated fatty acids like oleic acid, fatty acid, palmitic acid and stearic acid.^11^,^12^

The diet chocolate also showed a fall in their levels of pH but the fall in pH was lesser than both milk and dark chocolates. This could be due to the addition of components like aspartame and acesulfame K instead of sucrose, generally found to be anticariogenic,^13^ to prevent fall in pH^14^ and the adherence of plaque formed by mutans streptococci.^15^ Edgar and Dodds stated that the most beneficial action of these sweeteners is the stimulation of salivary flow and thus raising the pH.^16^

When the filled chocolates were compared, the chocolate with fruits and nuts showed the maximum drop in pH from the 15-30 minutes period (Graph 1). This could be due to the longer retention ability of the fillings and also could be attributed to the sucrose concentration of the fruits (in our study raisins were used) which additively makes it more acidogenic.

The chocolate with coconut fillings showed relatively lesser but significant fall in the pH. This could be due to its texture which enables it to be more retentive and therefore possibly more acidogenic. Moreover it has been seen that chocolate coated coconut cream caused about ten times as much enamel weight loss as did milk chocolate or caramel.^17^

The chocolates with caramel showed the least fall in pH making it the least acidogenic and cariogenic in the group. This could be due to the reason that chocolate caramel bars exhibited high initial retention rates and a very rapid rate of clearance from the teeth.^18^

When both the groups were compared, chocolate with fruit and nut had maximum drop in pH, which makes it the most cariogenic followed by the coconut chocolate and the least drop in pH was seen in the diet chocolate making it least cariogenic. Chocolate, particularly the dark chocolate (high cocoa content) and caramel (high initial retention and very rapid rate of clearance from the teeth) had a low acidogenic potential. Even diet chocolate was found to have a least drop in pH (because it contains an artificial sweetener which is found to be least cariogenic).

It should be remembered that chocolate is not entirely safe for the teeth, and frequent consumption by children of any food containing fermentable carbohydrates should be avoided. Consumed in sensible amounts, chocolate can be included in healthy eating, assuming the consumer is active and their diet is healthy and balanced.

## CONCLUSION

- Diet chocolate was found to have a least drop in plaque pH and found to be least cariogenic.
- Chocolate with fruit and nut was found to have a maximum pH drop, making it most cariogenic.
- The cariogenic potential of all filled chocolates are more cariogenic compared to unfilled chocolates.
